# Estimation Methods for Viscosity, Flow Rate and Pressure from Pump-Motor Assembly Parameters

**DOI:** 10.3390/s20051451

**Published:** 2020-03-06

**Authors:** Martin Elenkov, Paul Ecker, Benjamin Lukitsch, Christoph Janeczek, Michael Harasek, Margit Gföhler

**Affiliations:** 1 Institute of Engineering Design and Product Development, TU Wien, 1060 Vienna, Austria; paul.ecker@tuwien.ac.at (P.E.); christoph.janeczek@tuwien.ac.at (C.J.); margit.gfoehler@tuwien.ac.at (M.G.); 2 Institute of Chemical, Environmental and Bioscience Engineering, TU Wien, 1060 Vienna, Austria; benjamin.lukitsch@tuwien.ac.at (B.L.); michael.harasek@tuwien.ac.at (M.H.)

**Keywords:** blood pumps, flow rate, pressure difference, viscosity, estimation, Gaussian process regression

## Abstract

Blood pumps have found applications in heart support devices, oxygenators, and dialysis systems, among others. Often, there is no room for sensors, or the sensors are simply unreliable when long-term operation is required. However, control systems rely on those hard-to-measure parameters, such as blood flow rate and pressure difference, thus their estimation takes a central role in the development process of such medical devices. The viscosity of the blood not only influences the estimation of those parameters but is often a parameter that is of great interest to both doctors and engineers. In this work, estimation methods for blood flow rate, pressure difference, and viscosity are presented using Gaussian process regression models. Different water–glycerol mixtures were used to model blood. Data was collected from a custom-built blood pump, designed for intracorporeal oxygenators in an in vitro test circuit. The estimation was performed from motor current and motor speed measurements and its accuracy was measured for: blood flow rate r^2^ = 0.98, root mean squared error (RMSE) = 46 mL^.^min^−1^; pressure difference r^2^ = 0.98, RMSE = 8.7 mmHg; and viscosity r^2^ = 0.98, RMSE = 0.049 mPa^.^s. The results suggest that the presented methods can be used to accurately predict blood flow rate, pressure, and viscosity online.

## 1. Introduction

Positive-pressure mechanical ventilation is a highly unphysiological but widely used and needed mode of ventilation. Patients that undergo positive-pressure mechanical ventilation for extended periods of time often develop ventilator-induced lung injuries (VILI) and acute respiratory distress syndrome (ARDS) [1] which can lead to worse quality of life and even death. Usually, extracorporeal carbon dioxide removal (ECCO_2_R) [2] devices are combined with protective ventilation strategies to let the lungs recover. However, ECCO_2_R is a highly invasive procedure that is associated with elevated risks of infection, thrombosis and—due to the high priming volumes—high load on the heart [3]. An intracorporeal membrane oxygenator could be used as a supplementary mode of oxygenation along with mechanical ventilation and thus reduce the mechanical load on the lungs due to the high ventilator pressures. Additionally, an intracorporeal membrane oxygenator could help patients with sufficient lung capacity recover from various lung diseases and injuries by supplementing their spontaneous breathing.

Implantable rotary blood pumps have found wide applications in heart support or replacement [4], but can also be used as a tool to provide sufficient blood flow through membrane modules [5,6]. Intracorporeal membrane oxygenators introduce a non-physiological pressure drop at the site of implantation, thus increasing the load on the heart [7]. A miniaturized pump can compensate for the pressure drop created by the catheter and promote better gas exchange by guiding the blood through the fiber packing. It is desired that the pump is feedback-controlled, such as to follow physiological pressure differences—Δp and flow rates—*Q* thus preventing suction [8] and tissue damage and also regulate the gas exchange through the membrane catheter. However, due to the tendency of pressure sensors to drift and build thrombi at the site of the sensing diaphragm, and the difficulty of implementing a flow rate sensor [9] the control problem becomes more difficult. It would be advantageous if one could accurately estimate *Q* and Δp as a function of already available signals such as the current–*I* and the rotation speed–*ω* of the pump-motor assembly.

Various groups have already shown the feasibility of *Q* and Δp estimation for heart assist devices from other signals such as *ω* and motor power—*P* [10,11], *ω*, and motor torque—*τ* [12,13], *ω* and *I* [14,15,16,17,18], or even impeller radial displacement [19]. Almost all also considered the effects of blood viscosity *µ* [10,11,12,14,15,16,17,18,19,20]. Most of the efforts are focused on steady state estimation or averaging over many samples [10,11,19]. However, some efforts have been put into dynamic estimations with high bandwidth [15,16], e.g., Moscato et al. reported an root mean squared error (RMSE) of 252 mL^.^min^−1^ for the static and 444 mL^.^min^−1^ for the dynamic estimation of flow rate in water–glycerol mixtures. Most of the approaches employed a parametric polynomial model that was acquired by fitting the parameters to the measured characteristics of the device. The problem with that method is that an assumption about the underlying function of the process must be made. Furthermore, during operation often the characteristics of the system change—e.g., hematocrit levels and consequently viscosity, thus the estimation degrades with time. To combat that some groups have developed an online estimation of the viscosity of the blood from the pump-motor assembly parameters by either inducing vibrations in the radial direction [20,21], by occluding the pump exit [17] or by forcing the pump with a random signal for 10s every 2 min to get a frequency rich signal [18]. Hijikata et al. report their viscosity estimation methods with water–glycerol mixtures [21] (RMSE = 0.12 mPa^.^s in a range 1.18–5.12 mPa^.^s) and in vitro with blood [20] (RMSE = 0.12 mPa^.^s in a range 2.32–2.75 mPa^.^s). The inclusion of the viscosity compensation helped them improve the average error from 1830 mL^.^min^−1^ to 360 mL^.^min^−1^ in a range of 3000–5000 mL^.^min^−1^.

In this work, we propose a probabilistic non-parametric approach for estimation of *Q*, Δp, and *µ*, namely Gaussian process regression (GPR) [22]. GPR has the advantage over polynomial models of also providing a confidence interval together with the prediction. This additional information allows for more sophisticated decision making during the control of the system. The estimation algorithms were developed for a custom-built mixed-flow blood pump for an intracorporeal membrane oxygenator.

## 2. Materials and Methods

### 2.1. Data Collection and Data Processing

**Figure 1:**
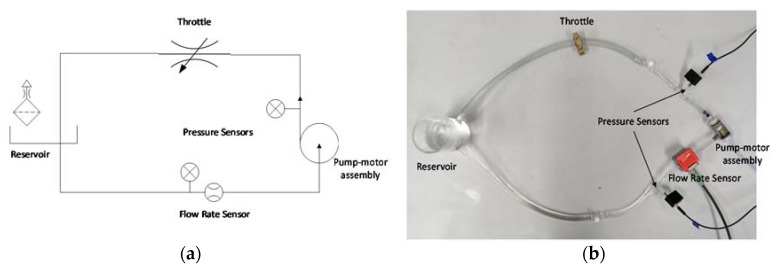
Test circuit: (**a**) Schematic; (**b**) Photograph.

The training and test data sets were acquired from a circulation model (Figure 1) consisting of the miniaturized rotary pump, a throttle that varies the hydraulic resistance of the system, absolute pressure sensors (AMS4711-2000-A, Analog Microelectronics, Mainz, Germany) before and after the pumping unit and an ultrasonic flow rate sensor (SonoFlow co.55, Sonotec, Halle, Germany). The system was supervised by a custom LabVIEW (National Instruments, Austin, Texas, USA) program and a real-time microcontroller (cRIO-9074, National Instruments, Austin, TX, USA). The data was stored with a sample time of $T_{s}=0,1\;s$. An analog low pass filter was implemented before the analog to digital converter stage with a cut-off frequency of 1.6 Hz. The custom-built 3D printed pumping unit was driven by an ECX-8 motor (Maxon Group, Sachseln, Switzerland). The data post-processing, evaluation of results and training and optimization of the GPR was done in MATLAB (MathWorks, Natick, MA, USA).

### 2.2. Viscosity and Blood Modeling

Water–glycerol mixtures in different proportions were used to model blood at different hematocrit levels and hence to represent different viscosity values. The mixture of water and glycerol is a Newtonian liquid, while blood is generally non-Newtonian and exhibits a shear-thinning behavior for shear rates < 200 s^−1^ [23]. However, rotary blood pumps usually develop shear rates in the order of 10^4^ s^−1^ [24], thus blood can be approximated as a Newtonian fluid with constant viscosity of 3.4 mPa^.^s in those shear rate ranges. The viscosity of different water–glycerol mixtures was measured and modeled by various groups [25]. It was shown by Cheng [26] that his formula has an average predicting error of 1.3% in a range of 0–100 °C and 0–100% water in the water–glycerol mixture. The analytic kinematic viscosity was thus calculated by:

(1) $$\nu =\nu_{1}^{\alpha }\nu_{2}^{\left(1-\alpha \right)},$$

where $\nu_{1}$ and $\nu_{2}$, are the kinematic viscosity of pure water and glycerol, respectively. The term α is a function of the mass fraction of glycerol $w_{2}$ and the temperature T of the solution defined as:

(2) $$\alpha =1-w_{2}+\frac{a\left(T\right)b\left(T\right)\left(1-w_{2}\right)w_{2}}{a\left(T\right)w_{2}+b\left(1-w_{2}\right)},$$

with a and b as polynomials of temperature:

(3) a=0.705−0.0017T,

(4) $$b=\left(4.9+0.036T\right)a^{2.5}$$

For ease of mixing with laboratory glass bottles, the mass fraction has been transformed to volume fraction through the density of the liquids. The kinematic viscosity can be transformed into dynamic viscosity by multiplying it with the density of the liquid. The kinematic and dynamic viscosity for the various water–glycerol mixtures used in this work can be seen in Table 1. The 65/35 water–glycerol volume percentage liquid mixture has approximately the same viscosity as blood with 40% hematocrit in its Newtonian regime.

### 2.3. Measurement Protocol

For the training data, the rotation speed of the pump was set at levels between 10000 and 30000 revolutions per minute (RPM) with a step size of 2500 RPM. For each RPM level, the hydraulic resistance of the system was varied by closing the throttle smoothly to produce monotonously falling flow rates in the range maximum flow rate Q_max_ to no flow rate Q_0_ and then monotonously rising flow rates Q_0_ to Q_max_ and their corresponding pressure drops *△p*. Additionally, training data consisting of combinations of throttle setting and rotations speed in random steps was acquired, in order to capture the dynamical behavior of the system. The *I*, *ω*, *Q*, *△p* quadruplets distributed over the whole operation range of the pump were recorded for 3 different test liquids (85/15, 65/35 and 50/50 water–glycerol volume percentage) with different viscosity (Table 1.). The test data set consisted of random combinations of *ω* and throttle setting and due to its randomness was different for each liquid mixture.

Additionally, a special type of signal, from here on referred to as the identification signal was recorded for each test liquid and was linked to its viscosity value. The identification signal consisted of 12 *I*, *ω* pairs and was recorded by doing an RPM sweep with a 2500 RPM step of the motor from 2500–30,000 RPM at open throttle and waiting for 1 s at each level. The identification signals were used to train a GP which linked each of the 12 *I*, *ω* pairs to the corresponding viscosity values of the liquid. A test set was also acquired for the evaluation of the model’s performance.

### 2.4. Gaussian Process Models

A Gaussian process [22] (GP) model of a real process f(x) is described fully by its mean function m(x) and covariance function $k\left(x_{i},x_{j}\right)$

(5) $$f\left(x\right)\;\sim \;GP\left(m\left(x\right),k\left(x_{i},x_{j}\right)\right).$$

The GP prior defines joint Gaussian distribution between the random variable vectors of the training inputs f(X) and the test inputs $f\left(X_{*}\right)$ i.e., the space of possible function values at training and test locations. The likelihood combines the observations y(X) with the random variable vector f(X) in a joint Gaussian distribution, essentially giving functions in f(X) that explain y(X) better a higher probability. The GP prior together with the likelihood form the Gaussian process model.

### 2.5. Gaussian Process Models Optimization

In this work, the mean and covariance functions, and the hyperparameters of the GPR models were optimized. Bayesian optimization method was used with the acquisition function expected improvement plus (EI+) [27]. Marginal likelihood was used as the objective function of the optimization process. The space of possible mean and covariance functions was spanned by:

- Mean Function: {none, constant, linear, quadratic}
- Covariance Function: {automatic relevance determination (ARD) exponential, ARD Matern kernel 3/2, ARD Matern kernel 5/2, ARD rational quadratic, ARD squared exponential, exponential, Matern kernel 3/2, Matern kernel 5/2, rational quadratic, squared exponential}

It was found that a pure quadratic mean function

(6) m(x)=Hβ

(7) $$H=\left[1,X_{1},X_{2}\right]$$

(8) $$X_{i}=\left(\begin{array}{ccc} x_{11}^{i} & \cdots & x_{1d}^{i}\\ \vdots & \ddots & \vdots \\ x_{n1}^{i} & \cdots & x_{nd}^{i} \end{array}\right),\;i=1,2$$

and ARD exponential function with custom length scales

(9) $$k\left(x_{i},x_{j}|\theta \right)=\sigma_{f}^{2}e^{-r}$$

(10) $$r=\sqrt{\overset{d}{\underset{m=1}{\sum }}\frac{{\left(x_{im}-x_{jm}\right)}^{2}}{\sigma_{m}^{2}}}$$

(11) $$\theta =\left(\sigma_{f}^{2},\sigma_{1}^{2},\ldots \sigma_{d}^{2}\right)$$

are the best performing for both flow rate and pressure estimation.

Finally, a custom GP model was developed from the data of each test liquid. The values of the hyperparameters θ (Equation (11)) were optimized for each one. In Equation (11) $\sigma_{f}^{2}$ is the estimated measurement noise, $\left(\sigma_{1}^{2},\ldots \sigma_{d}^{2}\right)$ are the custom length scales and the β (Equation (6)) is the coefficient vector of the mean function. Thus, the total number of optimization parameters is equal to D + N + 1, where D is the dimension of the input space, N is the polynomial degree of the mean function and +1 denotes the noise model parameter.

The described optimization process was done for the GPR model for both pressure and flow rate estimation. The flow rate estimation model and the pressure difference estimation model both took as input the last n = 2, *I*, *ω* values for a total of 4 inputs and produced as outputs the predicted flow rate—$\hat{Q}$ and the predicted pressure difference—$\hat{\Delta p}$ together with their respective standard deviations (SD).

The prediction is:

(12) $$f\left(X_{*}\right)=m\left(x\right)+K_{*}^{T}{\left(K_{y}^{}-\sigma_{f}^{2}I\right)}^{-1}\left(y-m\left(x\right)\right)$$

(13) $$cov\left(f_{*}\right)=K\left(X_{*}^{},X_{*}^{}\right)-K_{*}^{T}{\left(K_{y}^{}-\sigma_{f}^{2}I\right)}^{-1}K_{*}^{}+R_{}^{T}{\left(B_{}^{-1}+HK_{y}^{-1}H_{}^{T}\right)}^{-1}R,$$

with

(14) $$R=H_{*}-HK_{y}^{-1}K_{*}^{},$$

where B comes from β~N(b,B), $K_{*}^{}$ is the covariance matrix of the test and training set, $K_{y}^{}$ is the training set covariance matrix, $K\left(X_{*}^{},X_{*}^{}\right)$ is the test set covariance matrix, H is the training data polynomial matrix, $H_{*}$ is the test data polynomial matrix.

### 2.6. Viscosity Estimation GP

A separate GPR model was developed for estimating the viscosity of the test liquid. It was optimized for mean function and covariance function similar to the other GPR models. However, here the optimum was found at no mean function and Matern kernel 5/2 covariance function

(15) $$k\left(x_{i},x_{j}\right)=\sigma_{f}^{2}\left(1+\frac{\sqrt{5}r}{\sigma_{l}}+\frac{5r^{2}}{3\sigma_{l}^{2}}\right),$$

with,

(16) $$r=\sqrt{{\left(x_{i}-x_{j}\right)}^{T}\left(x_{i}-x_{j}\right)}.$$

This GPR model takes as an input the identification signal. The identification consists of 12 pairs of *I*, *ω* measurements at well-defined rotation speed values between 2500 RPM and 30,000 RPM in 2500 RPM steps for a total of 24 inputs.

### 2.7. Estimation Algorithm

**Figure 2:**
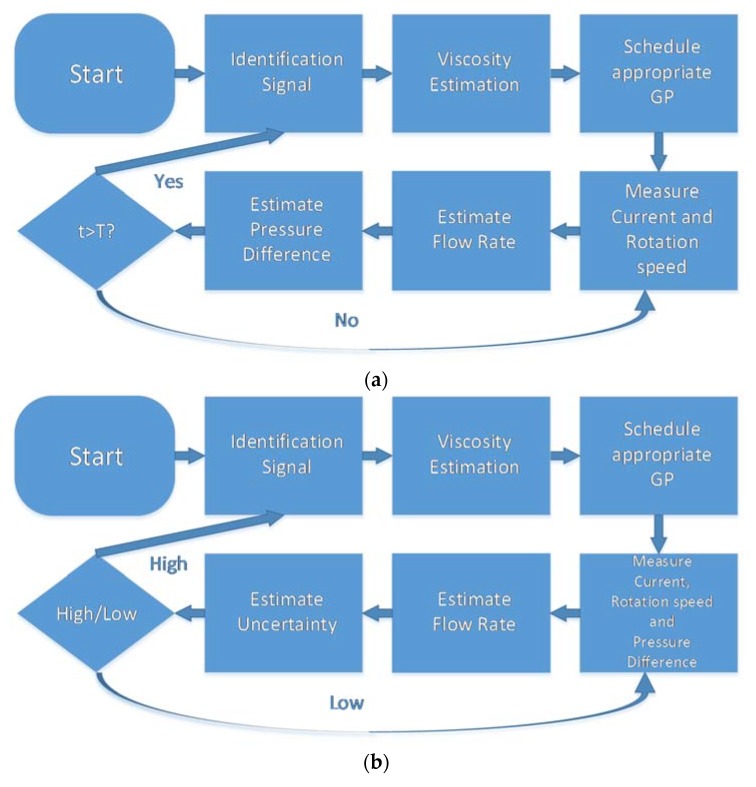
Flowchart of the two proposed algorithms for online estimation of hydrodynamic parameters. First the viscosity is automatically detected and the most suitable model for flow rate estimation and/or pressure becomes active. (**a**) The pressure and flow rate are continuously estimated with the same model as long as the time elapsed since the last viscosity identification is below a threshold. When the threshold time T elapses, the viscosity is re-identified; (**b**) The second algorithm needs an additional measurement signal to automatically detect when the viscosity should be re-identified. Here the pressure is measured along with the motor current and rotation speed and the flow rate is estimated. If the uncertainty of the estimation is above a threshold, the viscosity is re-identified. Otherwise, the flow rate is continuously estimated until the uncertainty becomes high.

Two algorithms for online estimation are proposed whose flowcharts are shown in Figure 2. For the first one (Figure 2a) the system starts by acquiring an identification signal which is then used to estimate the viscosity of the liquid. After that, a scheduling function enables the GP models which are most appropriate for the estimated viscosity levels. Then, the rotation speed and current of the motor are measured and used as inputs of the GP models that estimate the flow rate and the pressure difference. When a predefined time has elapsed, the identification signal is measured, and the viscosity is estimated to switch to the most appropriate GP model.

For the second algorithm (Figure 2b), the estimation of viscosity is not done in a predefined time interval, but instead, the uncertainty of the GP model is used as a clue as to when the prediction is wrong and the model needs to be changed. To do that, the GP models need additional information that differentiates them from each other, for example, a measurement of either pressure difference or flow rate. Since miniature pressure sensors are readily available, the pressure is measured along with the motor current and rotation speed and those are used to estimate the flow rate. A GP model was trained for this estimation algorithm, which took the three measured signals as input and estimated the flow rate. The uncertainty of the flow rate prediction would signal if the GP model needs to be changed and the viscosity re-identified.

## 3. Results

### 3.1. System Characterization

**Figure 3:**
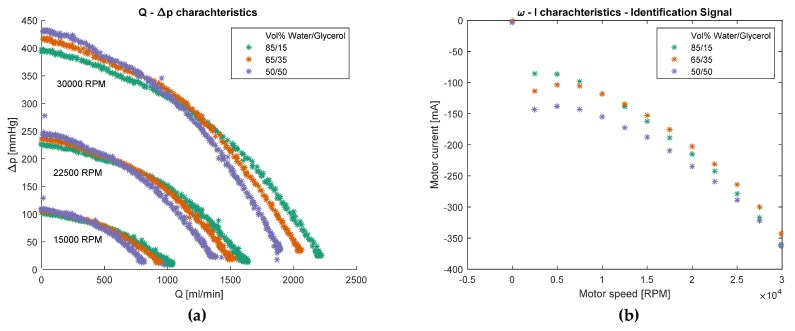
(**a**) *Q–△p* characteristics of the motor-pump assembly used in the experiment. Three clusters of curves can be seen corresponding to three different motor speeds, while the color coding refers to different viscosities of the test liquid; (**b**) *ω–I* characteristics of the motor - pump assembly. The motor current is plotted in a boxplot versus motor speed and viscosity of the test liquid.

As a first step of the system identification process, the characteristic curves of the custom-built pump were measured with different water–glycerol mixtures. Three clusters can be seen in the plot of Figure 3a, which corresponds to the *Q–△p* characteristics of the pump at different motor speeds—15,000, 22,500, and 30,000 RPM. The color coding corresponds to 85%, 65% and 50% water volume in the water–glycerol mixtures that have different viscosity. The *Q–△p* curves at equal motor speed start parallel at low flow rates. Then, after some viscosity dependent flow rate, the higher the viscosity the steeper the curves become. The *Q–△p* curves represent the hydrodynamic behavior of the system which is the part of the system to be estimated.

The *I–ω* characteristics or the so-called identification signal for different viscosities can be seen in the (b) plot of Figure 3. The pumping of the most viscous liquid requires the most current at low rotation speeds, while at high rotation speeds the curves seem to converge.

**Figure 4:**
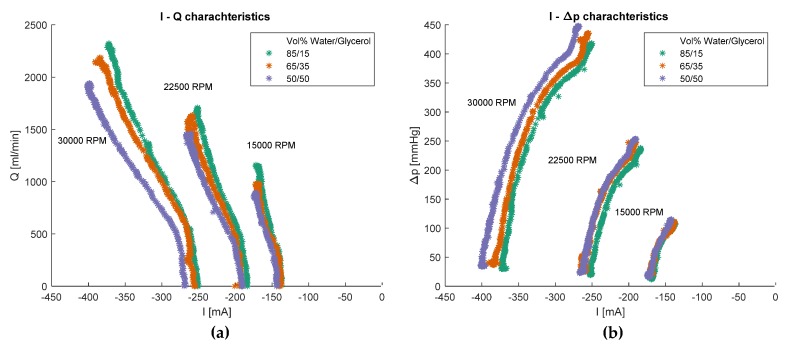
(**a**) The *I–Q* characteristics of the system; (**b**) The *I–△p* characteristics of the system. The three clusters of curves in both plots correspond to different motor speeds while the color coding refers to different viscosities of the test liquid.

Since we are trying to estimate flow rate and pressure difference from current measurements, it makes sense to look at what kind of curves describes the *I–Q* and *I–△p* relationships which are shown in Figure 4. The *I-Q* curves shown in the (b) plot of Figure 4 seem to start parallel and almost vertically for low currents. At high flows–high motor currents they start to diverge. All curves become flatter at higher motor currents, but the effect is more pronounced for higher viscosities. The almost vertical region at low currents and low flow rates suggest there could be low sensitivity of the models in this region. The *I–△p* curves on the (b) plot of Figure 4 seem to be parallel over the whole range of motor speeds and viscosities. For the same pressure difference, the system with more viscous liquid would require higher current. The results suggest that the curves are more separated at higher motor speeds, which is an effect that can be observed for all other characteristic curves except the *ω–I* curve in Figure 3b.

### 3.2. Estimating the Flow Rate and Pressure Difference

**Figure 5:**
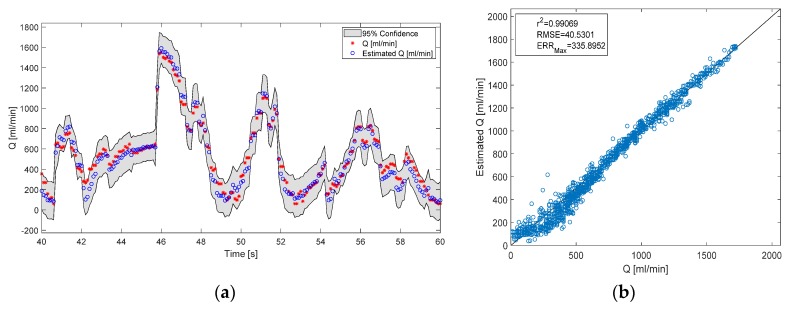
Results of the flow rate prediction. The estimation is done with a model that is trained on the same viscosity as the test signal. (**a**) Time series of the true and predicted flow rate with confidence intervals; (**b**) A scatter plot with fitness measures of the estimated vs. measured flow rate.

**Figure 6:**
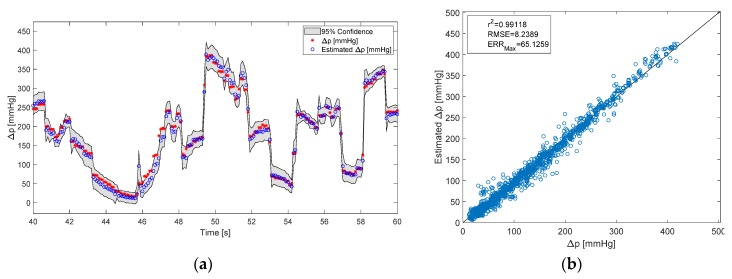
Results of the pressure difference prediction. The estimation is done with a model that is trained on the same viscosity as the test signal. (**a**) Time series of the true and predicted flow rate with confidence intervals; (**b**) A scatter plot with fitness measures of the estimated vs. measured pressure difference.

A GPR model was built for the estimation of both flow rate and pressure difference for each different test liquid. Then the fitness of the estimation $E_{ij}$ was evaluated for a test signal from each test liquid with different viscosity *i* = 3, estimated with each model trained on a different liquid *j* = 3 resulting in a total of *i* × *j* = 9 predictions. The mean correlation coefficient for all blood flow rate predictions $E_{ij}$, with *i* = *j*, was calculated at r^2^ = 0.98, while the RMSE = 46 mL^.^min^−1^ and the maximum error (definition see Appendix A below) ERR_max_ = 391 mL^.^min^−1^ (Figure 5) and for pressure difference (Figure 6) r^2^ = 0.98, RMSE = 8.7 mmHg and ERR_max_ = 67 mmHg. In comparison these values for the predictions $E_{ij}$, with *i* ≠ *j*, were: r^2^ = 0.94, RMSE = 236 mL^.^min^−1^, ERR_max_ = 555 mL^.^min^−1^ for blood flow rate and r^2^ = 95, RMSE = 29 mmHg, ERR_max_ = 109 mmHg for pressure difference.

### 3.3. Estimating the Viscosity of the Test Liquid

**Figure 7:**
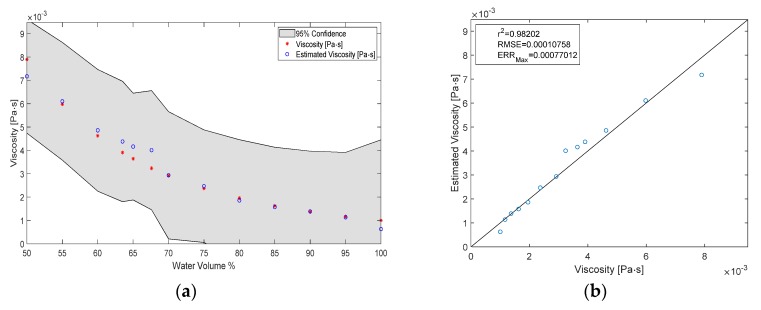
(**a**) Results of the viscosity estimation plotted versus volume fraction; (**b**) Fitness metrics of the estimation.

**Figure 8:**
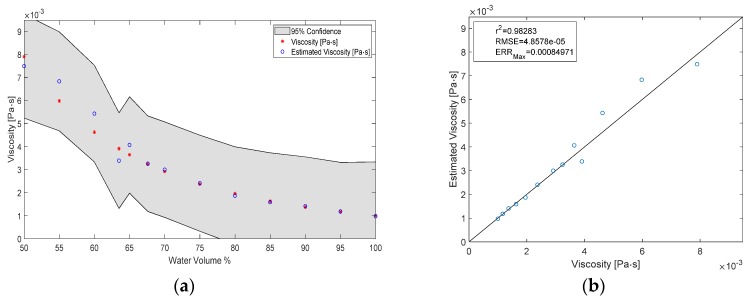
(**a**) Results of the viscosity estimation plotted versus volume fraction after inclusion of the 0.33 and 0.365 volume fraction points in the training process; (**b**) Fitness metrics of the estimation.

The viscosity of the liquid was estimated by sending the identification signal through the pump, measuring the electric current and motor speed and using those as inputs to a GPR model, which then predicted the viscosity. A result of the estimation can be seen in Figure 7 with some measures for fitness. All viscosity values at multiples of 5% volume have been part of the training of the model, while the liquids at 33% and 36.5% volume are unknown to the model. The overall correlation coefficient for the estimation of viscosity was r^2^ = 0.98, while RMSE = 0.11 mPa^.^s and ERR_max_ = 0.77 mPa^.^s. If the 33% and 36.5% water volume fraction liquids are also included in the training process, the estimation error improves to RMSE = 0.049 mPa^.^s. The results of the improved estimation can be seen in Figure 8.

### 3.4. Estimation of Uncertainty

**Figure 9:**
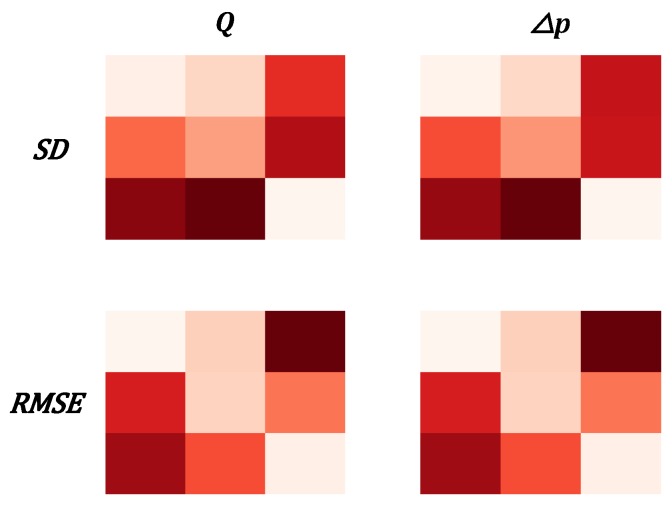
Illustrating the correlation between standard deviation and root mean squared error of the prediction. The left column of matrices shows the results for flow rate and the right column for pressure difference. The top row shows the matrices for standard deviation and the bottom row for RMSE. The matrices themselves show results for the 3 presented liquids thus far the 506,585 volume percentage water in the water–glycerol mixture. The diagonal values correspond to predictions $E_{ij}$, with *i* = *j*, while off-diagonal values correspond to $E_{ij}$, with *i* ≠ *j*. It can be seen that in both RMSE and standard deviation (SD) the diagonal values are much smaller than the off-diagonal values.

For the second proposed online estimation algorithm (Figure 2b) the uncertainty of the predictions signals the switching between GP models. The standard deviation of the prediction was estimated to be 26 mL^.^min^−1^ for *Q* and 5.5 mmHg for *△p* for $E_{ij}$, with *i* = *j*, and 31 mL^.^min^−1^ for *Q* and 6.2 mmHg for *△p* for $E_{ij}$, with *i* ≠ *j*. Figure 9 summarizes and visualizes those results and compares them to the RMSE. In all cases $E_{ij}$, with *i* = *j* the SD is lower compared to the $E_{ij}$, with *i* ≠ *j*, thus SD could be used as a signal that gives information about the error of the prediction model.

## 4. Discussion

In this work, the viscosity, flow rate, and pressure difference were estimated with the help of GPR models for a system consisting of a pump-motor assembly and various test liquids that model blood at different hematocrit levels with different viscosity.

The various system characteristic curves shown in Figure 3 and Figure 4 show distinct forms and suggest that there might be a unique mapping from the space of motor current and rotation speed to the space of pressure difference and blood flow rate. However, this mapping is unique only if a single test liquid is considered. As an example, at *I* = −300 mA and *ω* = 30,000 RPM the flow rate for the different liquids is approximately Q_50/50_ = 700 mL^.^min^−1^, Q_85/15_ = 950 mL^.^min^−1^, Q_65/35_ = 1050 mL^.^min^−1^, i.e., the mapping is not unique for the pump-motor assembly, but for the pump-motor-liquid system. Therefore, a separate GPR model was built for each liquid with different viscosity. Another possible solution is to train a single GPR model with an additional input parameter—the viscosity of the liquid. However, this approach is much more computationally expensive as the matrix inversion during the optimization/estimation process scales with ~*O*(*N*^3^). The non-uniqueness of the mapping also makes it necessary to include the additional signal measurement for the estimation algorithm in Figure 2b. Without it, the estimation could be completely wrong without any indication, since the motor current and rotation speed ranges are almost identical for all test liquids. At low rotation speeds, it is hard to distinguish between the characteristic curves of different test liquids, which is not a problem for high rotation speeds. Consequently, at a low rotation speed, even a wrong model would predict correctly. However, in this case, the controller would not know that the model is wrong since the SD of the prediction would also be low.

The estimation for the viscosities that were part of the training process is very accurate—RMSE = 0.07 mPa^.^s—even though the identification signal itself is new, and unknown to the model. However, the estimation of the viscosity of the new liquids is not as good—RMSE = 0.6 mPa^.^s—because the model has not been trained with data in that region. The viscosity estimation was improved by including more training points (Figure 9). This implies that the estimation accuracy can be improved further simply by introducing more data in a region of interest. For example, one might be interested in high estimation accuracy of blood viscosity with around 40% hematocrit. Then it would be sufficient if data is collected at several distinct hematocrit levels around the 40% mark and subsequently used to re-train the GPR model. The hydraulic resistance that the pump is exposed to does not change in our system, therefore the identification signal was always acquired in the same throttle position. This might not be the case for heart pumps.

A GP can be looked at as defining a radial basis function network (RBFN) with several basis functions equal to training data points and centered at each training data point [28]. Indeed, every arbitrary function can be expressed as a possibly infinite sum of radial basis function—i.e., RBFN is a universal approximator [29]. However, the advantage of a GP over an RBFN is that the number of optimization parameters is drastically reduced and in addition, the GP provides information about the certainty of the prediction. As discussed, the GPR model has D+N+1 optimization parameters while an RBFN would have to optimize for at least several parameters equal to the number of training points.

GP models output a Gaussian distribution, i.e., a mean and standard deviation, instead of a single value. This is a great advantage over non-probabilistic methods, e.g., polynomial models that always output a prediction even if that prediction is wildly incorrect. The level of uncertainty of the GPR prediction was used to automatically detect a shift in the viscosity of the test liquid.

One drawback of the GPR models, and in general all black box methods is that the model can never be more precise than the precision of the sensor used to collect the training data. Also, the model would learn and incorporate the biases of the sensor. The RMSE of the prediction for flow rate and pressure difference was measured at 46 mL^.^min^−1^ and 8.7 mmHg, respectively. The accuracy of the sensors is reported to be ~40 mL^.^min^−1^ and 7.5 mmHg for flow rate and pressure, respectively. Thus, it can be concluded that the presented models cover almost all the variability in the data and the uncertainty in the prediction is mostly of an aleatoric character. Additionally, there might be some bias in the test sets presented in this work, because the throttle was operated by hand, but the bias was in no way intended.

The order of the pressure estimation and flow rate estimation models was chosen to be 2 because it gave good correlation and small error between estimated and observed values. Additionally, a second-order model allows for the modeling of dynamic behavior such as time derivatives of input signals. However, that might not be the optimal order for the models. A more thorough analysis would be to train models with a larger amount of delayed inputs and choose the one, which has the best compromise between low objective function value and model complexity. In a sense of a polynomial model for statistical analysis of time series, the optimal order would correspond, according to Taken’s theorem [30], to less than 2d+1, where d is the order of the nonlinearity in the underlying process. A possible nonlinearity that was not modeled by the GPR of order 2 can be seen in the low flow rates regime in Figure 5b, where the scattered data deviates from the line of the perfect fit.

Another limitation is that instead of blood, various water–glycerol mixtures were used. Even though the shear rates produced by the pump are high enough so that blood can be approximated as a Newtonian liquid, there might be low-velocity zones in the flow profile, where the shear-thinning behavior of blood might come into play. However, in this work a Newtonian blood substitute was used, because the focus was more on the modeling of the system and proof of concept for the estimation of *Q*, *△p*, *µ*. Blood models that also exhibit shear-thinning behavior can be found in Campo-Deaño et al. [31].

A further limitation is that the density of the test liquids was not considered to be a variable during the modeling process. The pressure difference that the pump produces is

(17) △p=ρ·g·H,

with ρ the density of the liquid, g is the acceleration due to gravity and H is the pump pressure head. The effect of the density can be seen in Figure 3a, where for zero flow, the pressure difference developed by the pump is different for the different liquids. If instead H is used as a dependent variable in the estimation process, the model would become independent of ρ and all lines in Figure 3 would start at the same pressure head for zero flow. However, usually in physiological control applications, △p developed by the pump is of interest instead of H.

In conclusion, the feasibility of flow rate, pressure difference, and viscosity estimation from motor rotation speed and electric current was presented. The non-linear mappings from *ω–I* to *Q*, *△p*, *µ*, were modeled with GPR models, which were optimized for mean and covariance functions and hyperparameters. Blood with different hematocrit levels was modeled as different volume percentages of water–glycerol mixtures. The dynamic estimation of flow rate and pressure difference was very accurate (blood flow rate r^2^ = 0.98, RMSE = 46 mL^.^min^−1^; pressure difference r^2^ = 0.98, RMSE = 8.7 mmHg) and it was shown that it could be easily implemented for online use by including a viscosity measurement (r^2^ = 0.98, RMSE = 0. 0.049 mPa^.^s). In future work, the performance of the method will be tested in vitro with blood as a test liquid.

## Figures and Tables

**Table 1:** Kinematic and dynamic viscosities of different water–glycerol mixtures calculated analytically using Equations (1)–(4).

Water/Glycerol Vol%	100/0	95/5	90/10	85/15	80/20	75/25	70/30	65/35	60/40	55/45	50/50
Kinematic Viscosity [µm^2.^s^−1^] @23 °C	0.934	1.08	1.26	1.49	1.78	2.15	2.64	3.28	4.16	5.38	7.11
Dynamic Viscosity [µPa^.^s^−1^] @23 °C	1006	1156	1341	1571	1861	2231	2711	3344	4194	5359	6995

## Appendix A

### Measures of Fitness

The correlation coefficient (r^2^), the RMSE and the maximum error (ERR_max_) were used to evaluate the performance of the estimation. The r^2^ represents the extent to which the variance of the data is explained by the model. An r^2^ = 1, would suggest that all variance of the system is captured by the model, while r^2^ = 0, would suggest that the model is not explaining the underlying phenomena at all. The correlation coefficient r^2^, is defined as:

(A1) $$r^{2}=\frac{\sum_{i\in n}^{n}{\left({\hat{X}}_{i}-\bar{X}\right)}^{2}}{\sum_{i\in n}^{n}{\left(X_{i}-\bar{X}\right)}^{2}}$$

where ${\hat{X}}_{i}$ and $X_{i}$ are the estimated and measured variable, $\bar{X}$ is the mean of the measured variable and n is the number of observations. The RMSE is a measure of the mean error that we can expect from the prediction. The RMSE is defined as:

(A2) $$RMSE=\sqrt{\frac{\sum_{i\in n}^{n}{\left({\hat{X}}_{i}-X_{i}\right)}^{2}}{n}}$$

The maximum error that is observed during the prediction is captured by:

(A3) $${\mathrm{ERR}}_{max}=\underset{i\in n}{\max}\left(\sqrt{{\left({\hat{X}}_{i}-X_{i}\right)}^{2}}\right)$$

## References

[1] Beitler J.R., Malhotra A., Thompson B.T. (2017). Ventilator-Induced Lung Injury. Clin. Chest Med..

[2] Jeffries R.G., Lund L., Frankowski B., Federspiel W.J. (2017). An Extracorporeal Carbon Dioxide Removal (ECCO 2 R) Device Operating at Hemodialysis Blood Flow Rates. ICMx 5.

[3] Makdisi G., Wang I.W. (2015). Extra Corporeal Membrane Oxygenation (ECMO) Review of a Lifesaving Technology. J. Thorac. Dis..

[4] Matsuda H. (2000). Rotary Blood Pumps: New Developments and Current Applications.

[5] Cattaneo G., Strauß A., Reul H. (2004). Compact Intra- and Extracorporeal Oxygenator Developments. Perfusion.

[6] Cattaneo G.F.M., Reul H., Schmitz-Rode T., Steinseifer U. (2006). Intravascular Blood Oxygenation Using Hollow Fibers in a Disk-Shaped Configuration: Experimental Evaluation of the Relationship Between Porosity and Performance. ASIAO J..

[7] Thomson I.R. (1984). Cardiovascular Physiology: VENOUS RETURN. Can. Anaesth. Soc. J..

[8] Giridharan G.A., Skliar M. (2003). Control Strategy for Maintaining Physiological Perfusion with Rotary Blood Pumps. Artif. Organs.

[9] Bertram C.D. (2005). Measurement for Implantable Rotary Blood Pumps. Physiol. Meas..

[10] Lim E., Karantonis D.M., Reizes J.A., Cloherty S.L., Mason D.G., Lovell N.H. (2008). Noninvasive Average Flow and Differential Pressure Estimation for an Implantable Rotary Blood Pump Using Dimensional Analysis. IEEE Trans. Biomed. Eng..

[11] Malagutti N., Karantonis D.M., Cloherty S.L., Ayre P.L., Mason D.G., Salamonsen R.F., Lovell N.H. (2007). Noninvasive Average Flow Estimation for an Implantable Rotary Blood Pump: A New Algorithm Incorporating the Role of Blood Viscosity. Artif. Organs.

[12] Hijikata W., Rao J., Abe S., Takatani S., Shinshi T. (2015). Estimating Flow Rate Using the Motor Torque in a Rotary Blood Pump. Sens. Mater..

[13] Funakubo A., Ahmed S., Sakuma I., Fukui Y. (2002). Flow Rate and Pressure Head Estimation in a Centrifugal Blood Pump. Artif. Organs.

[14] Zhang X.T., Alomari A.H., Savkin A.V., Ayre P.J., Lim E., Salamonsen R.F., Rosenfeldt F.L., Lovell N.H. In Vivo Validation of Pulsatile Flow and Differential Pressure Estimation Models in a Left Ventricular Assist Device. Proceedings of the 2010 Annual International Conference of the IEEE Engineering in Medicine and Biology.

[15] Granegger M., Moscato F., Casas F., Wieselthaler G., Schima H. (2012). Development of a Pump Flow Estimator for Rotary Blood Pumps to Enhance Monitoring of Ventricular Function. Artif. Organs.

[16] Moscato F., Danieli G.A., Schima H. (2009). Dynamic Modeling and Identification of an Axial Flow Ventricular Assist Device. Int. J. Artif. Organs.

[17] Tsukiya T., Akamatsu T. (1997). Use of Motor Current in Flow Rate Measurement for the Magnetically Suspended Centrifugal Blood Pump. Artif. Organs.

[18] Kitamura T., Matsushima Y., Tokuyama T., Kono S., Nishimura K., Komeda M., Yanai M., Kijima T., Nojiri C. (2000). Physical Model-Based Indirect Measurements of Blood Pressure and Flow Using a Centrifugal Pump. Artif. Organs.

[19] Shida S., Masuzawa T., Osa M. (2019). Flow Rate Estimation of a Centrifugal Blood Pump Using the Passively Stabilized Eccentric Position of a Magnetically Levitated Impeller. Int. J. Artif. Organs.

[20] Hijikata W., Maruyama T., Suzumori Y. (2019). Measuring Real-Time Blood Viscosity with a Ventricular Assist Device. J. Eng. Med..

[21] Hijikata W., Rao J., Abe S., Takatani S., Shinshi T. (2015). Sensorless Viscosity Measurement in a Magnetically-Levitated Rotary Blood Pump. Artif. Organs.

[22] Rasmussen C.E., Williams C.K.I. (2006). Gaussian Processes for Machine Learning, 2006.

[23] Galdi G.P., Robertson A.M., Rannacher R., Turek S. (2008). Hemodynamical Flows.

[24] Fraser K.H., Taskin M.E., Griffith B.P., Wu Z.J. (2011). The Use of Computational Fluid Dynamics in the Development of Ventricular Assist Devices. Med. Eng. Phys..

[25] Gonzalez J.A.T., Longinotti M.P., Corti H.R. (2011). The Viscosity of Glycerol-Water Mixtures Including the Supercooled Region. J. Chem. Eng. Data.

[26] Cheng N.S. (2008). Formula for the Viscosity of a Glycerol-Water Mixture. Ind. Eng. Chem. Res..

[27] Snoek J., Larochelle H., Adams R.P. (2012). Practical Bayesian Optimization of Machine Learning Algorithms. Adv. Neural Inf. Process. Syst..

[28] Lightbody G., Gregorc G. (2009). Gaussian Process Approach for Modelling of Nonlinear Systems. Eng. Appli. Artif. Int..

[29] Park J., Sandberg I.W. (1991). Universal Approximation Using Radial-Basis-Function Networks. Neural Comput..

[30] Takens F., Takens F., Rand D., Young L.-S. (1981). Detecting Strange Attractors in Turbulence. Dynamical systems and turbulence, Warwick 1980.

[31] Campo-Deaño L., Dullens R.P.A., Aarts D.G.A.L., Pinho F.T., Oliveira M.S.N. (2013). Viscoelasticity of Blood and Viscoelastic Blood Analogues for Use in Polydymethylsiloxane In Iitro Models of the Circulatory System. Biomicrofluidics.

